# Angiographic description of the superior rectal artery and its anatomical variations in patients undergoing embolization of the superior rectal arteries in hemorrhoidal disease treatment

**DOI:** 10.31744/einstein_journal/2024AO0688

**Published:** 2024-09-20

**Authors:** Priscila Mina Falsarella, Marcelo Katz, Breno Boueri Affonso, Francisco Leonardo Galastri, Marcelo Froeder Arcuri, Joaquim Mauricio da Motta-Leal-Filho, Sérgio Eduardo Alonso Araujo, Rodrigo Gobbo Garcia, Felipe Nasser

**Affiliations:** 1 Hospital Israelita Albert Einstein São Paulo SP Brazil Hospital Israelita Albert Einstein, São Paulo, SP, Brazil.; 2 Hospital Municipal da Vila Santa Catarina Dr. Gilson de Cássia Marques de Carvalho Hospital Israelita Albert Einstein São Paulo SP Brazil Hospital Municipal da Vila Santa Catarina Dr. Gilson de Cássia Marques de Carvalho; Hospital Israelita Albert Einstein, São Paulo, SP, Brazil.

**Keywords:** Hemorrhoids, Embolization, therapeutic, Anatomic variation, Angiography, Mesenteric artery, inferior

## Abstract

**Objective:**

To describe angiographic findings of the superior rectal artery, its branches, and anatomical variations in the hemorrhoidal plexus in patients undergoing rectal artery embolization for hemorrhoidal disease treatment.

**Methods:**

Angiographic findings of 15 patients were obtained from a single-center, prospective clinical study that compared superior rectal artery embolization with the Ferguson technique for hemorrhoidal disease between July 2018 and March 2020.

**Results:**

Angiography of the superior rectal artery showed that in seven patients (46.8%), its branches were divided into four main branches (two left and two right), while in four patients (26.6%), the branches divided into one right and two left branches. The most uncommon variation observed in three cases (20%) was the branches divided into two branches to the right and one to the left; no further subdivision into the main branches was observed in one case (6.6%).

**Conclusion:**

Four patterns were observed in the angiographic anatomy of the superior rectal arteries. Knowledge of the angiographic anatomy of this region and its variations is essential to improve the effectiveness of superior rectal artery embolization.

**Registry of Clinical Trials:**

NCT03402282

## In Brief

Angiography of the superior rectal artery showed that its branches were divided into four main branches (two left and two right) in 46.8%; the second most frequent variation was one right and two left branches in 26.6%, followed by two branches to the right and one to the left in 20%; the most uncommon variations were one to the right and one to the left without further subdivision in 6.6%.

## Highlights

◼ The superior rectal artery, when it reaches the rectum, divides into two or more branches.
◼ Four patterns were observed in the angiographic anatomy of the superior rectal artery.
◼ Understanding the angiographic anatomy of the superior rectal artery is important to achieve optimal embolization results.

## Graphical abstract

**Figure:**
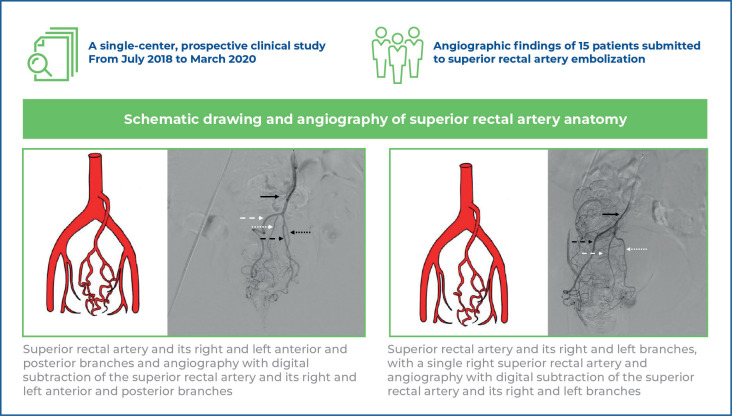

## INTRODUCTION

The prevalence of symptomatic hemorrhoidal disease is estimated to be 4-40%.^(1,2)^ The treatment indicated for patients with hemorrhoidal disease is clinical and can be surgical.^(3)^ Superior rectal artery embolization has emerged as a promising technique for treating hemorrhoidal disease.

In 1994, Galkin reported the first hemorrhoidal disease treatment through embolization of the branches of the superior rectal artery.^(4)^ Previous case series have demonstrated the efficacy of treating severe rectal bleeding from various causes through embolization of the superior rectal arteries.^(5–7)^ Recently, Vidal et al.^(8)^ described the "emborrhoid" technique, which consists of the superselective embolization of branches of the superior rectal arteries for internal hemorrhoidal disease treatment^(8)^ in patients with severe bleeding secondary to hemorrhoids disease and contraindication to conventional surgical treatment.^(2,8)^ After this initial experience, other series described the technical and clinical success of the "emborrhoid" technique in hemorrhoidal disease treatment.^(9–11)^ The anatomy of rectal arteries is not very complex; however, no study has described the angiographic findings of the superior rectal arteries and its branches related to hemorrhoidal disease. Understanding the number, distribution, frequency, branches, and potential anatomical variants of the rectal arteries can guide interventional radiologists, reducing the chance of technical and clinical failures in superior rectal artery embolization.

## OBJECTIVE

To describe the angiographic findings of the superior rectal artery, its branches, and anatomical variations in the hemorrhoidal plexus in patients undergoing rectal artery embolization for hemorrhoidal disease treatment.

## METHODS

The results described here are part of a single-center, prospective, randomized clinical trial that compared superior rectal artery embolization with microcoils and closed hemorrhoidectomy using the Ferguson technique.^(12)^ The study was approved by the institutional review board. Written informed consent was obtained from all the patients.

The inclusion criteria were internal grade 2 or 3 hemorrhoidal disease with clinical symptoms (such as pain, bleeding, mucus, pruritus, and prolapse), including indications for surgical treatment. The exclusion criteria were grade 4 internal hemorrhoids, external hemorrhoids, and contraindications to angiography.

The angiographies of 15 patients who underwent treatment for hemorrhoidal disease through superior rectal artery embolization between July 2018 and March 2020 were evaluated.

Hemorrhoid embolization procedures were performed under local anesthesia and sedation at the Interventional Medicine Center of our hospital by using the following angiographers: Philips Allura Xper FD20 (Philips Medical Systems Nederland), Philips Allura Clarity Xper FD20/10 biplane (Philips Medical Systems Nederland), and Henetix contrast (Iobitriol 350/ml, Guerbet^®^). Embolization of the rectal arteries was performed using 0.018 InterlockTM fiberized IDCTM fiber springs (Boston Scientific^®^).

The study was approved by the Research Ethics Committee of *Hospital Israelita Albert Einstein*, CAAE: 72364117.0.1001.0071; #2,653,722 and from the *Secretaria Municipal de Saúde de São Paulo*, CAAE: 72364117.0.3001.0086; 2,307,573.

### Technique

Catheterization with a 5F introducer (Super Sheath Boston Scientific^®^) was performed in the right common femoral artery, unilateral, followed by aortography with a 5F pigtail catheter (Infiniti Cordis^®^) at the level of L3 (third lumbar vertebra) to identify the origin of the inferior mesenteric artery and its branches. The pigtail catheter was changed to a 5F Simmons catheter curve 2 (5F Imager TM II Boston Scientific^®^). Selective catheterization of the inferior mesenteric artery was performed in the posteroanterior view, followed by angiography (arterial and venous phases - Figure 1 A and B), with identification of the superior rectal artery and a total volume of contrast of 12mL (4mL/sec and pressure of 300 psi). Then, using the road map technique, superselective catheterization of the superior rectal artery was performed with a RenegadeTM STC microcatheter and FathomTM −16 microguide (Boston Scientific^®^) and angiography with digital image subtraction in posteroanterior incidence with a total volume of 12mL of contrast (4mL/sec at a pressure of 700 psi) (Figure 1S, Supplementary Material).

**Figure 1:**
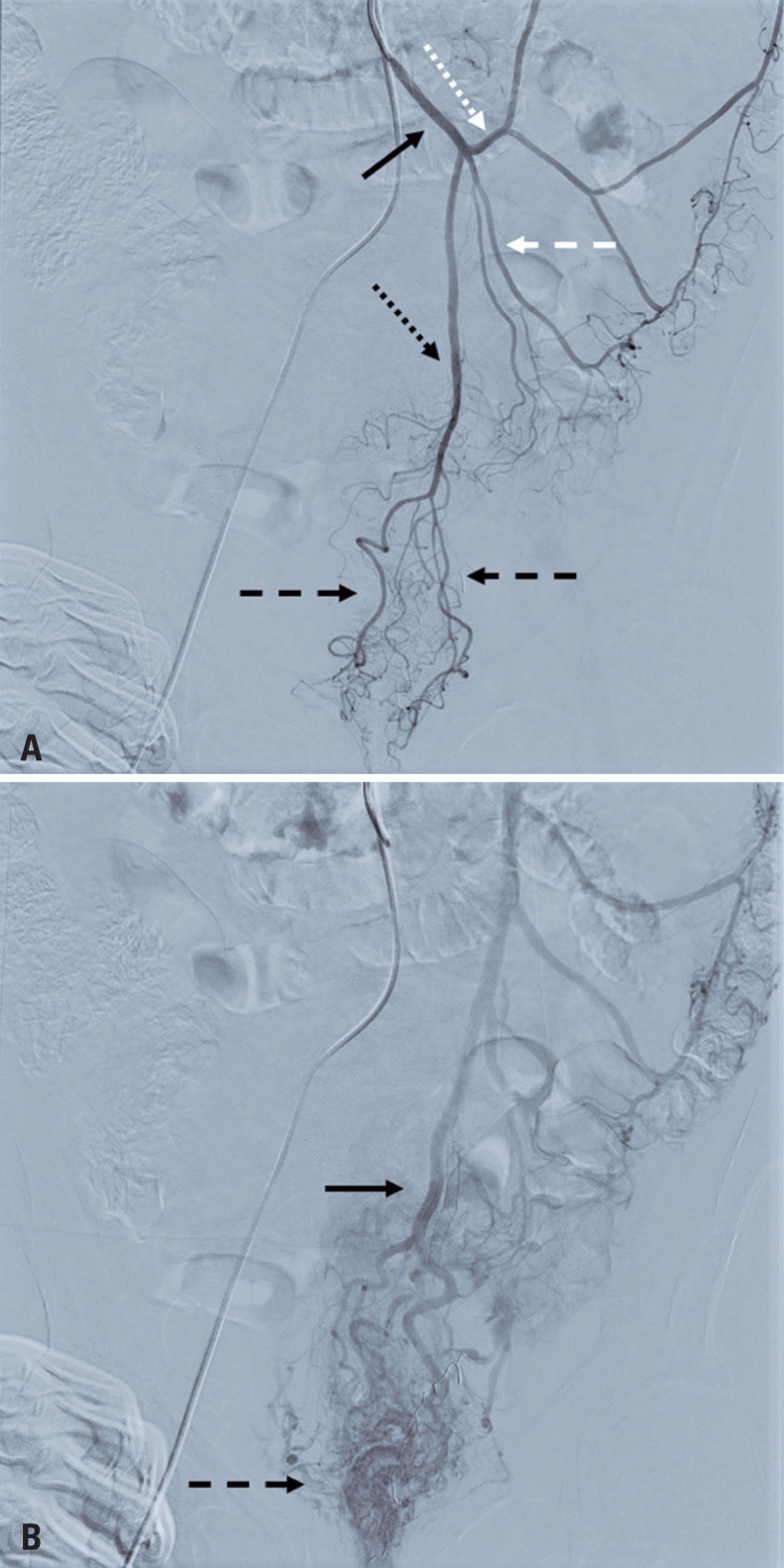
Angiography with digital subtraction of the inferior mesenteric artery. A) Angiography with digital subtraction (arterial phase) of the inferior mesenteric artery (black continuous arrow) demonstrating the superior rectal artery (black dotted arrow) and its right and left branches (black dashed arrows), sigmoid artery (white dashed arrow), and left colic artery (white dotted arrow); B) angiography with digital subtraction (venous phase) of the inferior mesenteric artery demonstrating the venous drainage of the hemorrhoidal plexus (dashed arrow) and superior rectal vein (continuous arrow)

The angiographies were reviewed together by two experienced interventional radiologists with more than 8 years of experience, and the distribution of the segmental branches of the superior rectal artery was described as a percentage.

## RESULTS

A total of 33 patients were included in the study: 29 underwent intervention for hemorrhoidal disease treatment, 15 underwent embolization with microcoils of the superior rectal arteries, 14 underwent surgical correction using the Ferguson closed hemorrhoidectomy technique, and four (one in the embolization group and three in the surgical group) were lost to follow-up before the intervention.

The embolization and surgery groups were statistically similar, with the embolization group consisting of nine males and six females, with a mean age of 54.6 years and a duration of hemorrhoidal symptoms of 7.83 years (similar to the surgery group).

**Figure 2:**
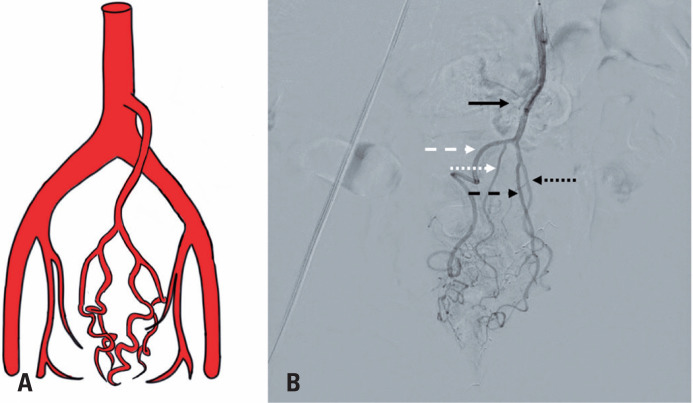
A) Schematic drawing of the superior rectal artery and its right and left anterior and posterior branches; B) Angiography with digital subtraction of the superior rectal artery and its right and left anterior and posterior branches; solid black arrow: superior rectal artery; white dotted arrow: right anterior branch; white dotted arrow: right posterior branch; black dashed arrow: left anterior branch; black dotted arrow: posterior left branch

**Figure 3:**
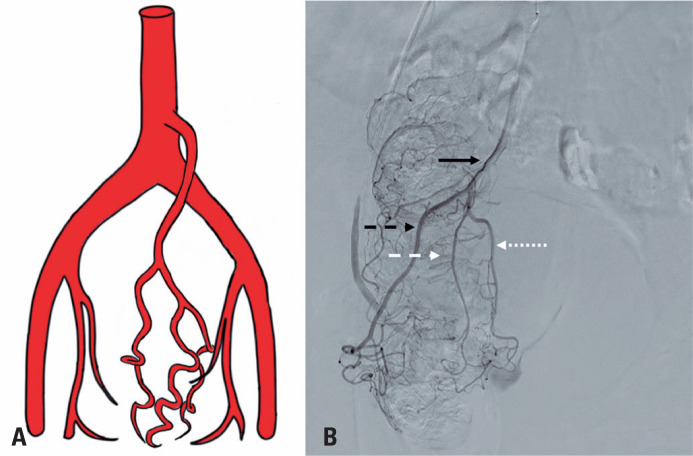
A) Schematic drawing of the superior rectal artery and its right and left branches, showing a single right superior rectal artery; B) Angiography with digital subtraction of the superior rectal artery and its right and left branches; solid black arrow: superior rectal artery; black dashed arrow: right branch; white dotted arrow: left anterior branch; white dotted arrow: posterior left branch

**Figure 4:**
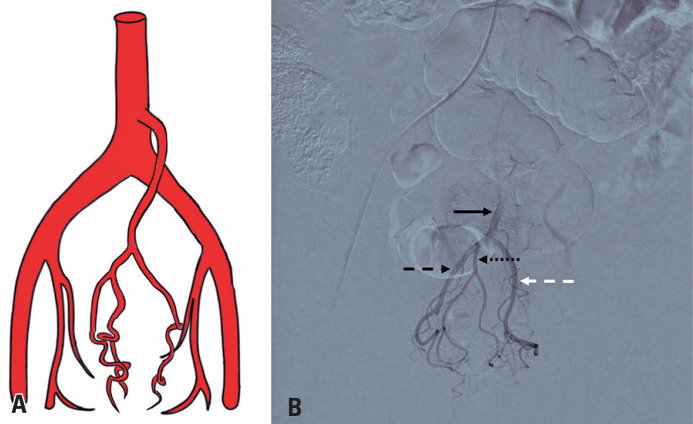
A) Schematic drawing of the superior rectal artery and its right and left branches, showing a single left superior rectal artery; B) Angiography with digital subtraction of the superior rectal artery and its right and left branches; solid black arrow: superior rectal artery; black dashed arrow: right anterior branch; black dotted arrow: right posterior branch; white dashed arrow: left branch

**Figure 5:**
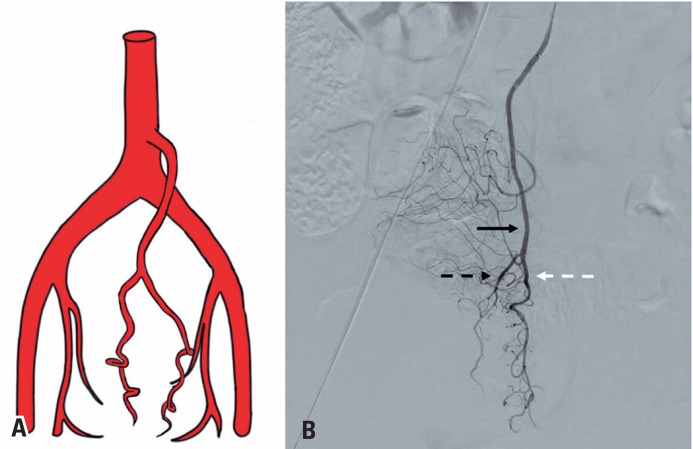
A) Schematic drawing of the superior rectal artery and its right and left branches, showing a single right and left superior rectal artery; B) Angiography with digital subtraction of the superior rectal artery and its right and left branches; solid black arrow: superior rectal artery; black dashed arrow: right branch; white dashed arrow: left branch

Among the 15 patients who underwent embolization, angiography of the superior rectal artery showed that its branches were divided into two to four main spiraled branches, with the division into right and left branches, with these being subdivided into anterior and posterior (generating four main branches) the usual anatomy, observed in seven patients (46.8% - Figures 2 A and B), the second most frequent variation was the division into right and left branches, with subsequent division of the left branch into anterior and posterior (totaling three main branches as hemorrhoid nourishers in four patients, 26.6% - Figures 3 A and B). Angiography showed two branches to the right (anterior and posterior) and one branch to the left in three cases (20%) (Figures 4 A and B), while subdivision into right and left branches, without further subdivision into main branches, was observed in one case (6.6 %) (Figures 5 A and B) (Table 1).

**Table 1:** Rectal artery branch distribution

Number of branches	Distribution	n (%)
4	right (A/P) + left (A/P)	7 (46.8)
3	right + left (A/P)	4 (26.6)
3	right (A/P) + left	3 (20)
2	right + left	1 (6.6)

The caliber of the branches ranged from 0.7mm to 3.1mm on the right (mean of 1.52 and median of 1.5mm); for the branches on the left, it ranged from 0.7 to 3.0mm (mean of 1.47 and median of 1.45mm).

## DISCUSSION

Internal hemorrhoids are nourished by branches of the inferior mesenteric artery, which begins a few centimeters proximal to the bifurcation of the aorta and originates the left colic, sigmoid, and superior rectal arteries that supply the proximal portion of the rectum.^(13)^

The superior rectal artery enters the muscular layer of the rectal mucosa below the third sacral vertebra.^(14)^ Once it reaches the rectum, it divides into two or more branches (right and left; the right branch is usually vicarious) that subdivide into smaller branches, forming loops around the lower rectum.^(13,14)^ In addition, it will have anastomoses with the middle rectal arteries (a direct branch of the anterior trunk of the internal iliac artery) and the inferior rectal artery (a branch of the internal pudendal artery).^(13)^

When the superior rectal artery reaches the rectum, it typically divides into two branches (right and left, the right being the more common one).^(2,15)^ In the present study, the most frequent anatomy observed in patients undergoing embolization (46.8%) was division into four branches (two on the right and two on the left, with no predominance between the right and left), followed by division into three branches (two left and one on the right) in 26.6% of the patients. The predominance of vascularization to the right (three branches, two on the right and one on the left) was observed in 20% of the patients.

Schuurman et al., in a study of the superior rectal artery in 10 cadavers without previous knowledge of hemorrhoidal disease, showed that the superior rectal artery divided into three to five branches, each spiraling around the rectum.^(16)^ The present study demonstrated the division into two to four branches with four different presentations of branch division.

Currently, some series have demonstrated the safety of the embolization technique of the superior rectal arteries in the treatment of internal hemorrhoidal disease.^(2,9,10,17)^ A more detailed knowledge of the angiographic anatomy of this region and its variations is vital for improving the method's effectiveness and reducing potential complications.

A limitation of this study was the small sample size due to the COVID-19 pandemic, which led to the interruption of outpatient clinics for benign proctological diseases at the institution.

## CONCLUSION

In conclusion, four patterns were observed in the angiographic anatomy of the superior rectal artery, with the most frequent distribution being the division of the rectal artery into four branches (two on the right and two on the left). Understanding the angiographic anatomy of this region and its variations is essential to improve the effectiveness of superior rectal artery embolization.

## Figures and Tables

**Figure 1S:**
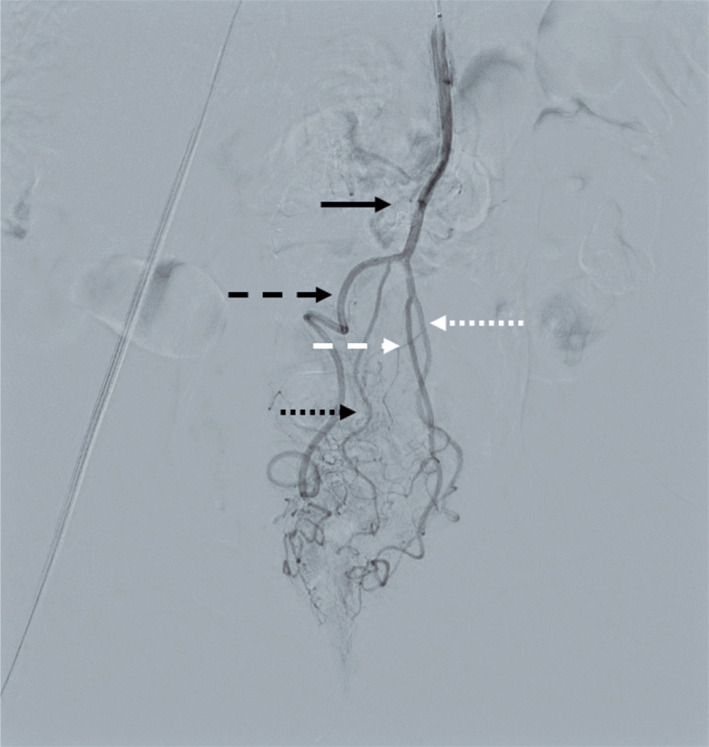
Superselective arterial phase digital subtraction angiography of the superior rectal artery and its right and left branches. Solid black arrow: superior rectal artery; black dashed arrow: right anterior branch; black dotted arrow: right posterior branch; white dotted arrow: left anterior branch; white dotted arrow: posterior left branch

## SUPPLEMENTARY MATERIAL

Angiographic description of the superior rectal artery and its anatomical variations in patients undergoing embolization of the superior rectal arteries in hemorrhoidal disease treatment

Priscila Mina Falsarella, Marcelo Katz, Breno Boueri Affonso, Francisco Leonardo Galastri, Marcelo Froeder Arcuri, Joaquim Mauricio da Motta-Leal-Filho, Sérgio Eduardo Alonso Araujo, Rodrigo Gobbo Garcia, Felipe Nasser

**DOI:** 10.31744/einstein_journal/2024AO0688
